# Sex-Specific Differences in Corneal Remodeling after Descemet Membrane Endothelial Keratoplasty in Fuchs Endothelial Corneal Dystrophy

**DOI:** 10.1016/j.xops.2026.101374

**Published:** 2026-08-19

**Authors:** Niloofar Kalantari, Gregor Lang, Sigrid Roters, Martine J. Jager, Berthold Seitz, Gerd Geerling, Sarah Barbara Zwingelberg

**Affiliations:** 1 Department of Ophthalmology, University Hospital of Düsseldorf (UoD), Düsseldorf, Germany; 2 Department for Functional Materials in Medicine and Dentistry, University Hospital of Würzburg, Würzburg, Germany; 3 Department of Ophthalmology, University Hospital of Cologne (UoC), Cologne, Germany; 4 Department of Ophthalmology, Leiden University Medical Center, Leiden, The Netherlands; 5 Department of Ophthalmology, Saarland University, Medical Center, Homburg/Saar, Germany

**Keywords:** Fuchs endothelial corneal dystrophy (FECD), Descemet membrane endothelial keratoplasty (DMEK), Corneal remodeling, Peripheral corneal thickness, Biologic sex

## Abstract

**Objective/Purpose:**

To evaluate sex-specific differences in preoperative disease severity and postoperative outcomes, including endothelial cell count (ECC), central corneal thickness (CCT), peripheral corneal thickness (PCT), and visual acuity, in patients with Fuchs endothelial corneal dystrophy (FECD) undergoing Descemet membrane endothelial keratoplasty (DMEK).

**Design:**

Retrospective, single-center cohort study.

**Participants/Subjects:**

Of the 181 eyes screened, 144 eyes (95 women, 49 men) with FECD undergoing DMEK at a tertiary referral center met the inclusion criteria of complete clinical data and ≥12 months of postoperative follow-up.

**Main Outcome Measures:**

Best spectacle-corrected visual acuity (BSCVA, logarithm of the minimum angle of resolution), ECC, CCT, and PCT assessed preoperatively and at 1, 3, 6, and 12 months postoperatively.

**Results:**

Mean (standard deviation) age at surgery was 72.8 (9.6) years in women and 70.9 (10.1) years in men (P = 0.25). Preoperatively, men had lower ECC (1466 ± 362 vs. 1729 ± 370 cells/mm²; *P* = 0.003), greater CCT (747.0 ± 148.0 vs 678.3 ± 128.8 µm; *P* = 0.014), and greater PCT (782.4 ± 105.1 vs. 702.6 ± 65.1 µm; *P* < 0.001), while BSCVA was comparable (*P* = 0.26). Following DMEK, both sexes showed similar visual improvement and CCT normalization. At 12 months, BSCVA (*P* = 0.26) and CCT (*P* = 0.98) did not differ significantly, whereas men had lower ECC (1487 ± 330 vs. 1727 ± 415 cells/mm²; *P* = 0.004) and greater PCT (722.6 ± 71.7 vs. 681.5 ± 66.3 µm; P = 0.015). In multivariable analysis, male sex was independently associated with greater 12-month PCT (*P* < 0.001), but not with 12-month ECC after adjustment for baseline disease severity and donor-related factors.

**Conclusions:**

Men with FECD exhibited more pronounced preoperative structural corneal changes and greater PCT after DMEK despite comparable visual recovery. Biological sex remained independently associated with postoperative PCT, but not ECC, after adjustment. These findings suggest sex-specific differences in FECD structural manifestations and postoperative corneal remodeling and warrant validation in prospective multicenter studies.

Fuchs endothelial corneal dystrophy (FECD) is the leading indication for endothelial keratoplasty in industrialized countries and represents the most common primary disorder of the corneal endothelium. Progressive endothelial cell dysfunction results in impaired corneal deturgescence, leading to stromal edema, increased corneal thickness, loss of transparency, and ultimately visual impairment. As human corneal endothelial cells possess only limited proliferative capacity in vivo, endothelial cell loss is primarily compensated through enlargement and migration of the remaining cells rather than cellular regeneration.^1^, ^2^, ^3^, ^4^, ^5^, ^6^

Descemet membrane endothelial keratoplasty (DMEK) has become the current standard of care for visually significant FECD, providing excellent visual rehabilitation, rapid recovery, low rejection rates, and long-term graft survival. Nevertheless, postoperative outcomes remain heterogeneous. Although most patients achieve substantial improvements in visual acuity and central corneal thickness (CCT), considerable variability exists in endothelial cell survival and structural corneal remodeling after transplantation.^7^, ^8^, ^9^, ^10^, ^11^, ^12^, ^13^ The factors contributing to this variability are still incompletely understood.

Fuchs endothelial corneal dystrophy pathogenesis is multifactorial and has been associated with endothelial cellular senescence, epigenetic alterations, and systemic and sociodemographic risk factors.^14^, ^15^, ^16^, ^17^ One of the most consistent epidemiological observations in FECD is its marked female predominance, with women being affected approximately twofold to fourfold more frequently than men.^18^, ^19^, ^20^, ^21^ Despite this well-established sex difference in disease prevalence, considerably less is known about whether biological sex is associated with differences in disease severity at presentation or influences postoperative recovery after DMEK. Previous studies have primarily focused on conventional outcome measures such as visual acuity, graft survival, and endothelial cell count (ECC), whereas potential sex-specific differences in corneal structural remodeling have received comparatively little attention.

wIncreasing evidence suggests that conventional clinical parameters alone may not fully reflect the structural consequences of endothelial dysfunction. Although CCT is widely used as a marker of corneal edema, peripheral corneal thickness (PCT) may provide complementary information regarding the spatial distribution of corneal hydration and postoperative tissue remodeling. Likewise, ECC reflects only 1 aspect of endothelial health and should be interpreted together with structural corneal parameters. Whether these morphological markers differ between female and male patients after DMEK has not previously been systematically investigated.

Improved understanding of sex-specific differences may have important clinical implications. If male and female patients differ not only in disease prevalence but also in structural disease severity or postoperative corneal remodeling, biological sex should be considered when interpreting surgical outcomes and designing future clinical studies. Furthermore, identifying structural biomarkers that complement conventional outcome measures may improve postoperative monitoring and provide a more comprehensive assessment of endothelial keratoplasty.

Therefore, the aim of this retrospective cohort study was to compare preoperative disease characteristics and postoperative functional and structural outcomes after DMEK between female and male patients with FECD. Specifically, we evaluated sex-specific differences in best spectacle-corrected visual acuity (BSCVA), ECC, CCT, and PCT during a 12-month follow-up period.

## Methods

### Study Design and Patient Selection

This retrospective, single-center cohort study included consecutive patients with FECD who underwent DMEK, either as isolated DMEK or combined with cataract surgery (triple DMEK), at the Department of Ophthalmology, University Hospital of Cologne.

A total of 181 consecutive DMEK procedures performed for FECD were screened for eligibility. Eyes were eligible if they had a confirmed diagnosis of FECD, underwent successful DMEK, and had complete preoperative examinations together with a minimum postoperative follow-up of 12 months. Thirty-seven eyes were excluded because of incomplete 12-month follow-up or missing clinical data, resulting in a final study cohort of 144 eyes of 144 patients, including 95 women and 49 men. Because of the retrospective study design, no a priori sample size calculation was performed. The characteristics of the excluded patients are summarized in Table 1.

**Table 1:** Patient Selection and Excluded Cases

Selection Step/Characteristic	Number of Eyes	Follow-Up Available	Comment
Consecutive DMEK procedures for FECD screened	181	Variable	Screened according to the predefined eligibility criteria
Excluded eyes	37	No complete 12-month follow-up and/or missing clinical data	Excluded before final analysis
Included eyes in final cohort	144	Complete 12-month follow-up	95 female and 49 male patients
Sex distribution among excluded patients	18 (m):19 (w)	Variable	Excluded cases were not retained in the final analysis spreadsheet
Age of excluded patients	70.3 ± 9.6 (m) vs. 71.2 ± 9.4 (w)	Variable	Excluded cases were not retained in the final analysis spreadsheet
Reason for exclusion	37	Incomplete follow-up and/or missing clinical data	Consistent with the predefined exclusion criteria described in Methods

DMEK = Descemet membrane endothelial keratoplasty; FECD = Fuchs endothelial corneal dystrophy.

The uploaded analysis dataset contains the final included cohort only. The 37 excluded patients had already been removed according to the predefined exclusion criteria.

Eyes with ocular comorbidities that could independently affect visual outcomes or corneal morphology were excluded. These included age-related macular degeneration, diabetic retinopathy, diabetic macular edema, advanced glaucoma, amblyopia, retinal diseases, previous corneal transplantation, glaucoma surgery, vitreoretinal surgery, and incomplete clinical documentation.

The study adhered to the tenets of the Declaration of Helsinki and was approved by the local institutional review board at the University of Cologne (approval no. 14-373). Owing to the retrospective study design and the use of anonymized routinely collected clinical data, the requirement for individual informed consent was waived according to institutional regulations.

### Clinical Assessment

Demographic and clinical variables included biological sex, age at surgery, surgical procedure (isolated DMEK or triple DMEK), donor age, donor ECC, postoperative rebubbling, BSCVA, recipient ECC, CCT, and PCT.

Clinical examinations were performed preoperatively and at 1, 3, 6, and 12 months after surgery. Best spectacle-corrected visual acuity was recorded in decimal notation and converted to the logarithm of the minimum angle of resolution (logMAR) for statistical analysis.

Recipient ECC was measured using noncontact specular microscopy (EM-3000, Tomey). Three consecutive measurements were obtained at each visit, and the mean value was used for analysis. Donor ECC was determined at the eye bank before transplantation by manual analysis of specular microscopy images.

Central corneal thickness and PCT measurements were obtained using Scheimpflug tomography (Pentacam, Oculus Optikgeräte GmbH). For the purposes of this study, PCT was operationally defined as the mean corneal thickness measured at a radial distance of 6.0 mm from the corneal vertex in the superior, inferior, nasal, and temporal quadrants. Thus, PCT represents a composite 4-quadrant measurement at the 6.0-mm measurement ring rather than a single peripheral location. The mean of these 4 quadrant-specific measurements was used for all statistical analyses.

Postoperative graft detachment requiring rebubbling was defined as clinically significant graft separation involving the visual axis or more than one-third of the graft area, confirmed by slit-lamp biomicroscopy and anterior segment OCT (Spectralis, Heidelberg Engineering). The indication for rebubbling followed standardized institutional criteria and was determined by the treating corneal surgeon.

### Surgical Technique

All DMEK procedures were performed by experienced corneal surgeons (Prof. Dr Björn Bachmann and Prof. Dr Claus Cursiefen) using previously published standardized surgical techniques.^7^, ^8^, ^9^, ^10^, ^11^, ^12^ Depending on the lens status, patients underwent either isolated DMEK or combined cataract surgery with DMEK (triple DMEK).

Donor Descemet membrane grafts measuring approximately 7.5–8.5 mm in diameter were prepared immediately before transplantation.

Recipient descemetorhexis was routinely performed under air with a diameter of approximately 7.5-8.0 mm, according to institutional standards.

Peripheral laser iridotomy was performed 1 day before surgery to reduce the risk of postoperative pupillary block.

Following graft implantation, the anterior chamber was filled with 20% sulfur hexafluoride gas for graft attachment. Patients were instructed to maintain a supine position for 72 hours postoperatively.

Postoperative treatment consisted of topical prednisolone acetate 1% in a tapering regimen over 24 months, topical antibiotic therapy for 1 week, pilocarpine 2% during the period of gas tamponade, and preservative-free artificial tears throughout follow-up.

### Statistical Analysis

Statistical analyses were performed using IBM SPSS Statistics version 25.0 (IBM Corp). Continuous variables are presented as mean ± standard deviation. Normality was assessed using the Kolmogorov–Smirnov test.

Baseline comparisons between female and male patients were performed using the independent Student *t* test or the Mann–Whitney *U* test, as appropriate. Categorical variables, including rebubbling rates, were compared using Fisher exact test.

Longitudinal postoperative changes in BSCVA, ECC, CCT, and PCT were analyzed using linear mixed-effects models, including time, sex, and the time × sex interaction as fixed effects, with patient-specific random intercepts to account for repeated measurements.

To identify factors independently associated with postoperative structural outcomes, complete–case multivariable linear regression analyses were performed using 12-month ECC and 12-month PCT as dependent variables. Independent variables included biological sex, age at surgery, surgical procedure (isolated DMEK versus triple DMEK), donor age, donor ECC, baseline ECC, and baseline CCT. Regression coefficients are reported as unstandardized β estimates with 95% confidence intervals (CIs).

Spearman rank correlation coefficients were calculated to evaluate associations between ECC, CCT, and PCT. Secondary univariable comparisons and correlation analyses were considered exploratory. Given the number of secondary comparisons performed, no formal adjustment for multiplicity was applied; corresponding *P* values should therefore be interpreted descriptively and with appropriate caution.

Missing values were not imputed, and all analyses were performed using available–case data. All statistical tests were 2-sided, and a *P* value <0.05 was considered statistically significant.

## Results

### Study Population

A total of 181 consecutive eyes undergoing DMEK for FECD were screened for eligibility. Thirty-seven eyes were excluded because of incomplete 12-month follow-up and/or missing clinical data, resulting in a final study cohort of 144 eyes of 144 patients, including 95 women (66.0%) and 49 men (34.0%) (Table 1). The mean follow-up period was 17.9 ± 12.6 months (range, 12–36 months).

The mean age at diagnosis was 68.2 ± 9.7 years in women and 66.4 ± 10.4 years in men (*P* = 0.30). Likewise, age at surgery did not differ significantly between female and male patients (72.8 ± 9.6 vs. 70.9 ± 10.1 years, *P* = 0.25). Isolated DMEK was performed in 36 of 95 women (38%) and 18 of 49 men (37%), whereas the remaining patients underwent triple DMEK.

Donor characteristics were comparable between both groups. Mean donor age was 65.0 ± 11.5 years in the female group and 67.2 ± 10.0 years in the male group (*P* = 0.24). Likewise, donor ECC did not differ significantly (2703 ± 203 vs. 2754 ± 216 cells/mm^2^, *P* = 0.18) (Table 2).

**Table 2:** Baseline Characteristics and Longitudinal Clinical Outcomes of Female and Male Patients with Fuchs Endothelial Corneal Dystrophy (FECD) Undergoing Descemet Membrane Endothelial Keratoplasty (DMEK)

Variable	Female (n = 95)	Male (n = 49)	*P* Value
Baseline characteristics			
Age at surgery, years	72.8 ± 9.6	70.9 ± 10.1	0.25
Donor age, years	65.0 ± 11.5	67.2 ± 10.0	0.24
Donor endothelial cell count, cells/mm^2^	2703 ± 203	2754 ± 216	0.18
Best spectacle-corrected visual acuity (logMAR)			
Preoperative	0.38 ± 0.22	0.45 ± 0.34	0.26
1 month	0.32 ± 0.28	0.21 ± 0.18	**0.007**
3 months	0.17 ± 0.18	0.14 ± 0.14	0.34
6 months	0.09 ± 0.08	0.09 ± 0.07	0.90
12 months	0.06 ± 0.07	0.07 ± 0.08	0.26
Endothelial cell count (cells/mm^2^)			
Preoperative	1729 ± 370	1466 ± 362	**0.003**
1 month	1840 ± 348	1766 ± 343	0.35
3 months	1782 ± 348	1653 ± 393	0.17
6 months	1756 ± 338	1609 ± 446	0.22
12 months	1727 ± 415	1487 ± 330	**0.004**
Central corneal thickness (μm)			
Preoperative	678.3 ± 128.8	747.0 ± 148.0	**0.014**
1 month	593.6 ± 102.7	586.6 ± 60.6	0.75
3 months	566.3 ± 72.8	595.4 ± 58.5	0.13
6 months	541.8 ± 43.5	554.0 ± 56.7	0.41
12 months	541.0 ± 47.0	540.7 ± 37.5	0.98
Paracentral/peripheral corneal thickness (μm)			
Preoperative	702.6 ± 65.1	782.4 ± 105.1	**<0.001**
1 month	817.7 ± 113.7	835.3 ± 127.5	0.50
3 months	740.8 ± 82.6	767.1 ± 72.5	0.19
6 months	718.5 ± 63.8	723.3 ± 71.7	0.79
12 months	681.5 ± 66.3	722.6 ± 71.7	**0.015**

logMAR = logarithm of the minimum angle of resolution.

Continuous variables are presented as mean ± standard deviation. *P* values represent comparisons between female and male patients at each time point.

Statistically significant differences are shown in bold.

### Visual Outcomes

Preoperative BSCVA did not differ significantly between female and male patients (0.38 ± 0.22 vs. 0.45 ± 0.34 logMAR, *P* = 0.26). Visual acuity improved substantially after DMEK in both groups throughout follow-up.

At 1 month, male patients demonstrated slightly better BSCVA than female patients (0.21 ± 0.18 vs. 0.32 ± 0.28 logMAR, *P* = 0.007). However, no statistically significant differences were observed at 3 months (*P* = 0.34), 6 months (*P* = 0.90), or 12 months (0.07 ± 0.08 vs. 0.06 ± 0.07 logMAR, *P* = 0.26), indicating comparable long-term visual rehabilitation after DMEK (Table 2, Fig 1).

**Figure 1:**
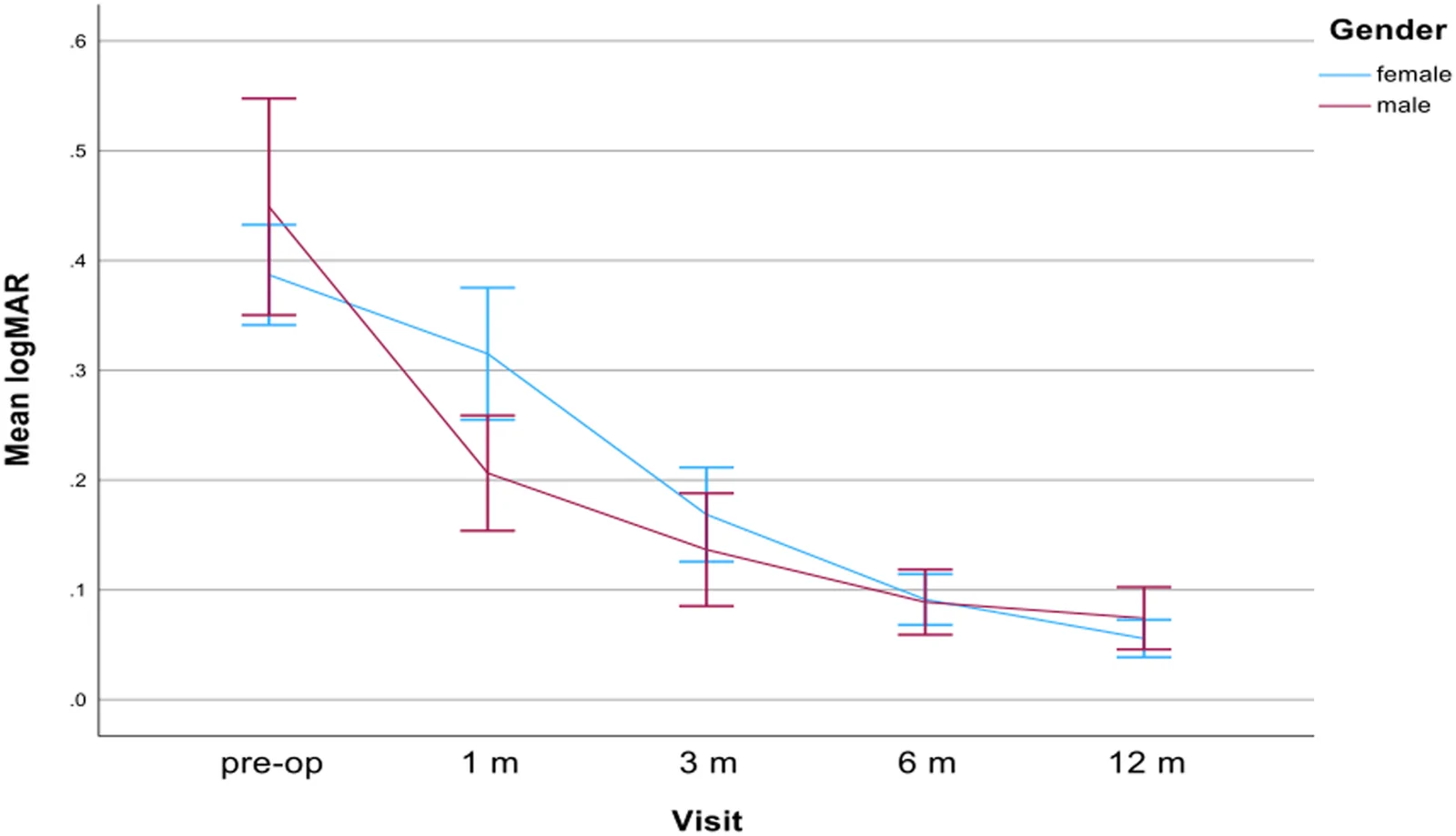
Longitudinal changes in best spectacle-corrected visual acuity (BSCVA) after Descemet membrane endothelial keratoplasty in female and male patients with Fuchs endothelial corneal dystrophy. Mean BSCVA (logMAR) was assessed preoperatively and at 1, 3, 6, and 12 months after surgery. Error bars represent 95% confidence intervals. Asterisks indicate statistically significant differences between female and male patients at individual time points (∗*P*∗ < 0.05, ∗∗*P*∗ < 0.01, and ∗∗∗*P*∗ < 0.001). logMAR = logarithm of the minimum angle of resolution.

### ECC

Endothelial cell count was significantly lower in male patients than in female patients before surgery (1466 ± 362 vs. 1729 ± 370 cells/mm^2^, *P* = 0.003).

After DMEK, ECC gradually decreased in both groups. Although no significant differences were observed at 1 month (*P* = 0.35), 3 months (*P* = 0.17), or 6 months (*P* = 0.22), male patients continued to exhibit significantly lower ECC at 12 months (1487 ± 330 vs. 1727 ± 415 cells/mm^2^, *P* = 0.004) (Table 2, Fig 2). Among eyes with available postoperative ECC measurements, the overall mean endothelial cell loss was 49.6 ± 11.0%, corresponding to a median cell loss of 48.4% (range, 33.6%–67.7%). No significant difference in endothelial cell loss was observed between female and male patients (49.0 ± 11.2% vs. 50.8 ± 10.7%, *P* = 0.43).

**Figure 2:**
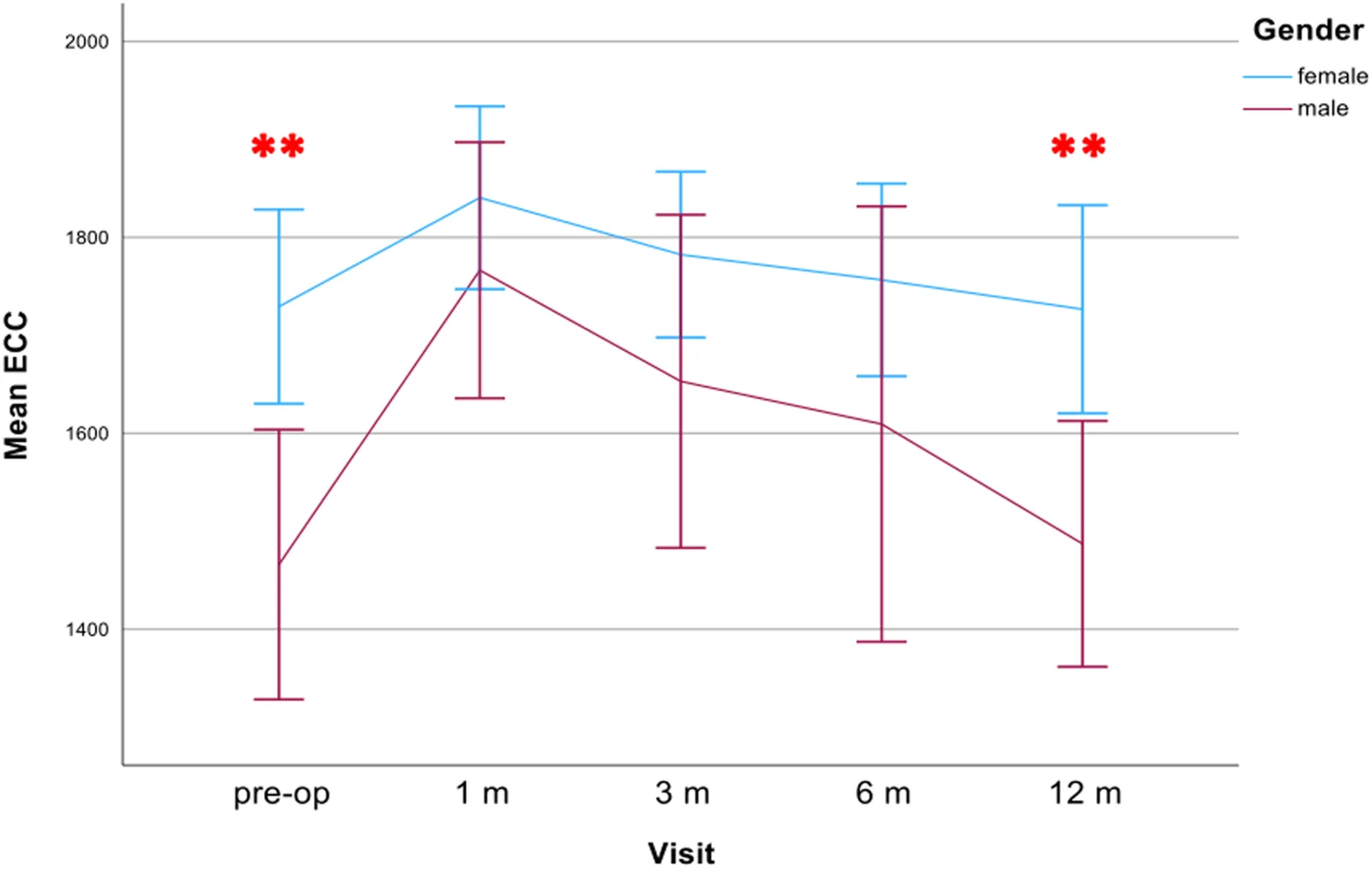
Longitudinal changes in endothelial cell count (ECC) after Descemet membrane endothelial keratoplasty in female and male patients with Fuchs endothelial corneal dystrophy. Mean ECC (cells/mm^2^) was assessed preoperatively and at 1, 3, 6, and 12 months after surgery. Error bars represent 95% confidence intervals. Asterisks indicate statistically significant differences between female and male patients at individual time points (∗*P*∗ < 0.05, ∗∗*P*∗ < 0.01, and ∗∗∗*P*∗ < 0.001).

### CCT

Preoperatively, CCT was significantly greater in male patients than in female patients (747.0 ± 148.0 vs. 678.3 ± 128.8 μm, *P* = 0.014).

After DMEK, CCT decreased markedly in both groups, with no statistically significant differences observed at 1 month (*P* = 0.75), 3 months (*P* = 0.13), 6 months (*P* = 0.41), or 12 months (540.7 ± 37.5 vs. 541.0 ± 47.0 μm, *P* = 0.98), indicating comparable restoration of CCT after surgery (Table 2, Fig 3).

**Figure 3:**
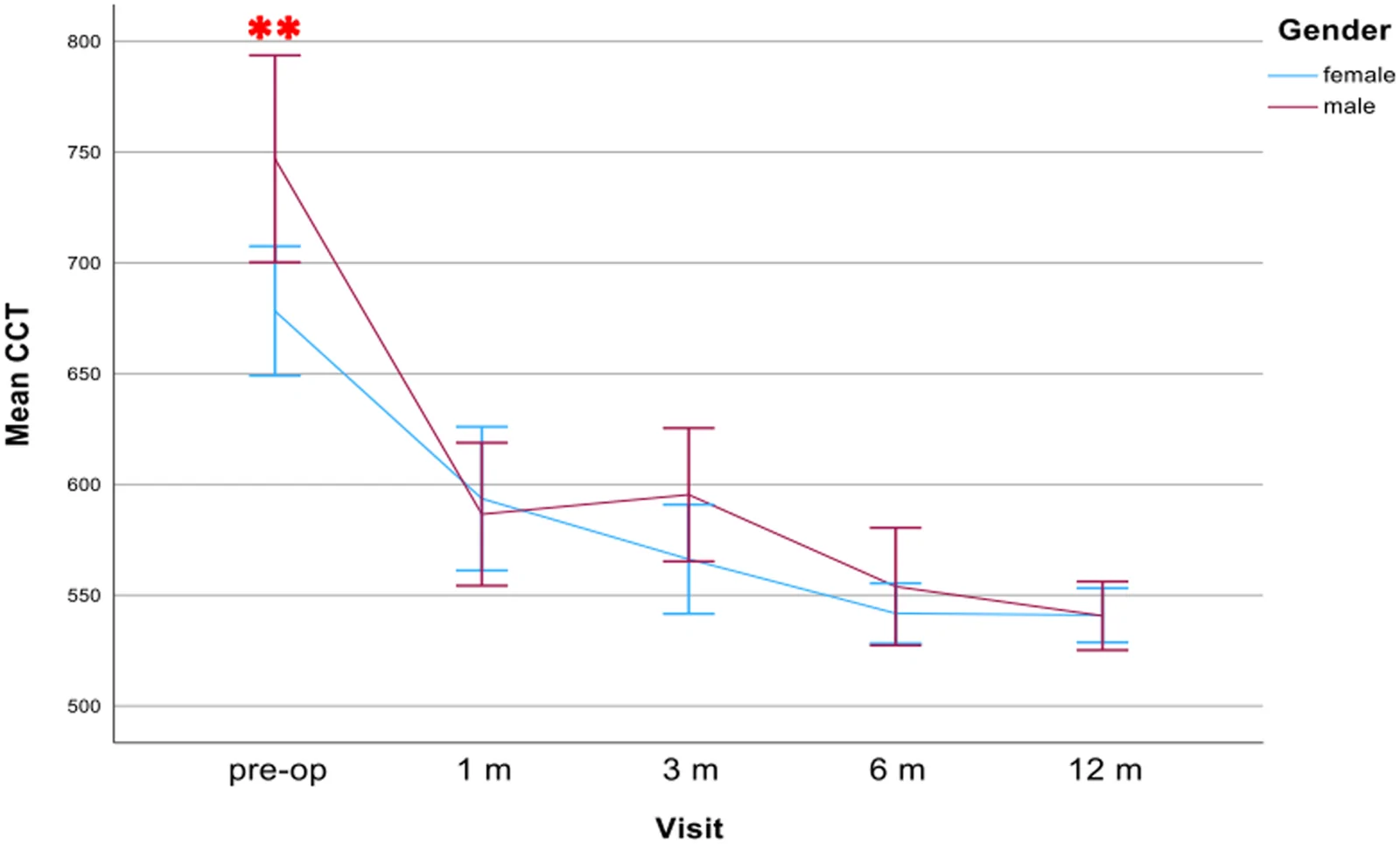
Longitudinal changes in central corneal thickness (CCT) after Descemet membrane endothelial keratoplasty in female and male patients with Fuchs endothelial corneal dystrophy. Mean CCT (μm) was measured preoperatively and at 1, 3, 6, and 12 months after surgery. Error bars represent 95% confidence intervals. Asterisks indicate statistically significant differences between female and male patients at individual time points (∗*P*∗ < 0.05, ∗∗*P*∗ < 0.01, and ∗∗∗*P*∗ < 0.001).

### PCT

Male patients presented with significantly greater PCT before surgery (782.4 ± 105.1 vs. 702.6 ± 65.1 μm, *P* < 0.001).

Peripheral corneal thickness decreased substantially after DMEK in both groups. No statistically significant differences were observed at 1 month (*P* = 0.50), 3 months (*P* = 0.19), or 6 months (*P* = 0.79). However, male patients continued to demonstrate significantly greater PCT at 12 months (722.6 ± 71.7 vs. 681.5 ± 66.3 μm, *P* = 0.015) (Table 2, Fig 4).

**Figure 4:**
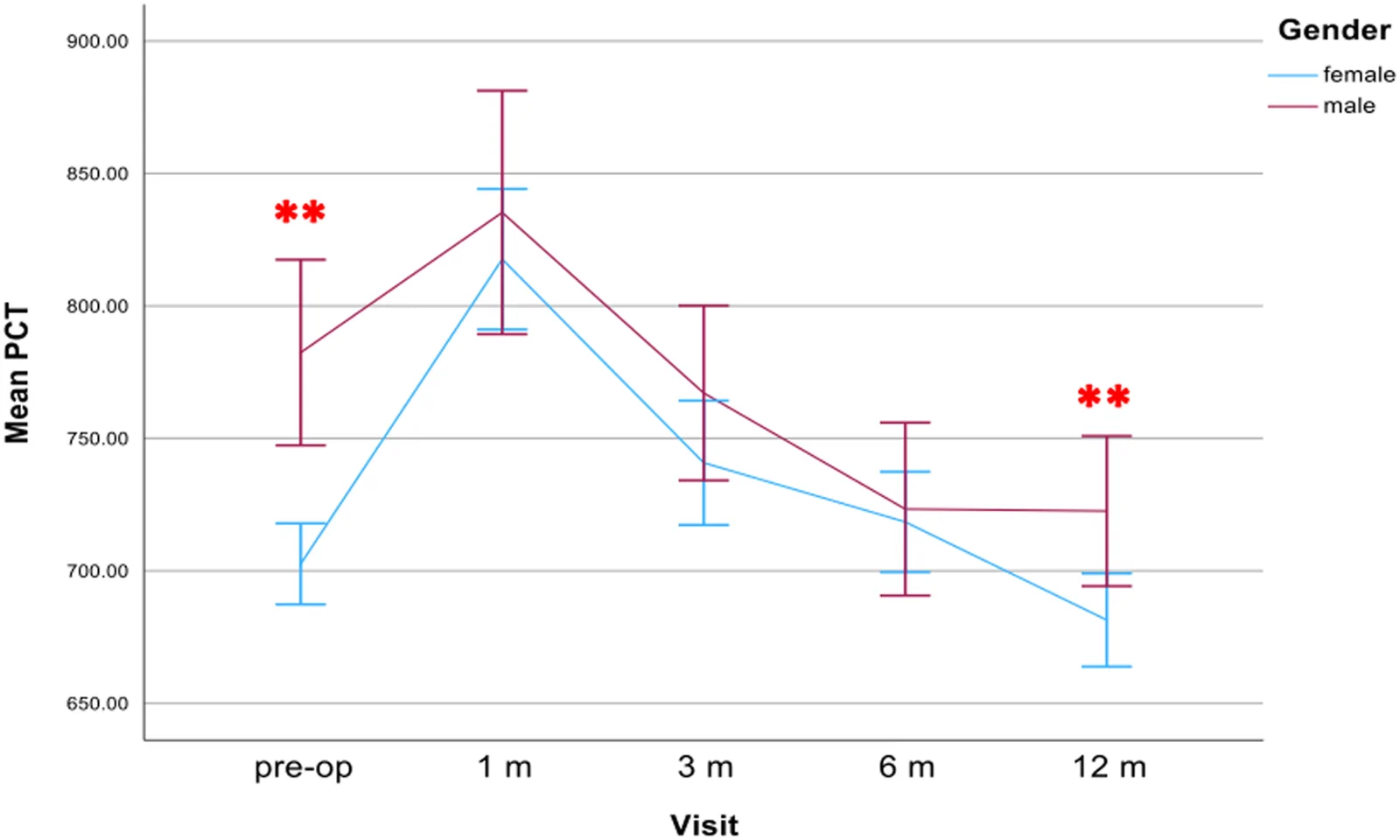
Longitudinal changes in peripheral corneal thickness (PCT) after Descemet membrane endothelial keratoplasty in female and male patients with Fuchs endothelial corneal dystrophy. Mean PCT (μm), measured 6.0 mm from the corneal vertex, was assessed preoperatively and at 1, 3, 6, and 12 months after surgery. Error bars represent 95% confidence intervals. Asterisks indicate statistically significant differences between female and male patients at individual time points (∗*P*∗ < 0.05, ∗∗*P*∗ < 0.01, and ∗∗∗*P*∗ < 0.001).

### Longitudinal Analysis

Linear mixed-effects models demonstrated significant longitudinal changes in BSCVA, CCT, and PCT over the postoperative follow-up period (all *P* < 0.001). In contrast, the overall time effect for ECC was not statistically significant (*P* = 0.19).

Significant sex × time interactions were observed for BSCVA (*P* = 0.009), ECC (*P* = 0.033), CCT (*P* = 0.006), and PCT (*P* = 0.012), indicating that the postoperative trajectories differed between female and male patients for both functional and structural outcome measures (Table 3).

**Table 3:** Linear Mixed-Effects Models Evaluating Longitudinal Postoperative Changes in Functional and Structural Outcome Measures after DMEK

Outcome	Time Effect	Time × Sex
BSCVA	<0.001	0.009
ECC	0.19	0.033
CCT	<0.001	0.006
PCT	<0.001	0.012

BSCVA = best spectacle-corrected visual acuity; CCT = central corneal thickness; DMEK = Descemet membrane endothelial keratoplasty; ECC = endothelial cell count; PCT = paracentral/peripheral corneal thickness.

Linear mixed-effects models were used to assess longitudinal postoperative changes in BSCVA, ECC, CCT, and paracentral/PCT. Fixed effects included time, biological sex, and the time × sex interaction, with patient-specific random intercepts to account for repeated measurements. *P* values are shown for the overall effect of time and the time × sex interaction.

### Correlation Analysis

No significant correlations were observed between PCT and ECC at 12 months in the overall cohort or after sex stratification.

In contrast, a moderate positive correlation between CCT and ECC was observed in male patients (Spearman ρ = 0.483, *P* = 0.027), whereas no significant association was identified in female patients.

### Multivariable Linear Regression Analyses

To identify factors independently associated with postoperative structural outcomes, multivariable linear regression analyses were performed using 12-month ECC and 12-month PCT as dependent variables (Table 3).

For postoperative ECC, donor ECC remained the only independent predictor of higher 12-month ECC (β = 72.6 per 100 cells/mm^2^; 95% CI, 17.8 to 127.3; *P* = 0.011). Male sex was not independently associated with postoperative ECC after adjustment for age at surgery, surgical procedure, donor characteristics, baseline ECC, and baseline CCT (β = –188.3; 95% CI, –443.4 to 66.9; *P* = 0.144). These findings indicate that the lower postoperative ECC observed in the univariable analysis was no longer evident after adjustment for baseline disease severity and donor-related factors. The model explained 21.5% of the variance in 12-month ECC (adjusted R^2^ = 0.215).

For postoperative PCT, male sex remained independently associated with greater PCT (β = 92.0 μm; 95% CI, 43.3 to 140.8; *P* < 0.001), whereas triple DMEK was independently associated with lower postoperative PCT (β = –64.2 μm; 95% CI, –114.7 to –13.7; *P* = 0.014).

No significant independent associations were observed for age at surgery, donor age, donor ECC, baseline ECC, or baseline CCT. The model explained 25.7% of the variance in postoperative PCT (adjusted R^2^ = 0.257) (Table 4).

**Table 4:** Multivariable Linear Regression Models Evaluating Factors Associated With 12-Month Endothelial Cell Count and Paracentral/Peripheral Corneal Thickness after DMEK

Predictor	12-month ECC (Cells/mm^2^)			12-Month PCT (μm)		
	β	95% CI	*P* Value	β	95% CI	*P* Value
Male sex	–188.3	–443.4 to 66.9	0.144	92.0	43.3 to 140.8	**<0.001**
Age at surgery, per year	–6.9	–19.3 to 5.5	0.265	–1.2	–3.5 to 1.2	0.322
Triple DMEK	–131.2	–408.5 to 146.2	0.345	–64.2	–114.7 to –13.7	**0.014**
Donor age, per year	–9.6	–20.5 to 1.3	0.084	0.3	–1.9 to 2.6	0.771
Donor ECC, per 100 cells/mm^2^	72.6	17.8 to 127.3	**0.011**	–7.9	–17.9 to 2.1	0.116
Baseline ECC, per 100 cells/mm^2^	22.5	–13.6 to 58.6	0.215	4.0	–2.9 to 10.9	0.25
Baseline CCT, per 10 μm	–0.5	–8.3 to 7.3	0.893	–0.3	–1.7 to 1.1	0.643
Adjusted R^2^	0.215			0.257		

CCT = central corneal thickness; CI = confidence interval; DMEK = Descemet membrane endothelial keratoplasty; ECC = endothelial cell count; PCT = paracentral/peripheral corneal thickness.

Male sex was coded as male vs. female. Triple DMEK was coded as triple DMEK vs. phakic DMEK. Missing values were not imputed.

### Postoperative Complications

Rebubbling was required in 6 female patients (6.3%) and in no male patients. This difference did not reach statistical significance according to Fisher exact test (*P* = 0.096). No additional sex-specific differences in postoperative complications were observed.

## Discussion

The present study evaluated sex-specific differences in patients with FECD undergoing DMEK. Three principal findings emerged. First, male patients presented with significantly lower ECC and greater CCT and PCT despite comparable preoperative visual acuity. Second, DMEK resulted in comparable long-term visual rehabilitation and normalization of CCT in both sexes. Third, male patients continued to demonstrate lower ECC and greater PCT at 12 months after surgery. However, multivariable analysis revealed that biological sex was independently associated with postoperative PCT but not with postoperative ECC after adjustment for baseline disease severity and donor-related factors.

These findings highlight an important distinction between functional and structural outcome measures in FECD.

Although BSCVA remains the primary clinical endpoint after endothelial keratoplasty, it does not necessarily reflect the extent of endothelial dysfunction or corneal remodeling. In the present cohort, women and men achieved comparable postoperative visual outcomes despite persistent differences in ECC and PCT. This observation suggests that structural imaging biomarkers may provide complementary information beyond visual acuity when assessing disease severity and postoperative tissue remodeling.

Notably, male patients exhibited a more pronounced structural disease phenotype at presentation, despite the well-established higher prevalence of FECD among women.^18^, ^19^, ^20^, ^21^ The reasons underlying this observation remain uncertain.

One possible explanation is delayed presentation or referral, as sex-related differences in health care utilization and symptom perception have been described in several chronic diseases. Alternatively, biological differences in endothelial susceptibility, hormonal regulation, oxidative stress, or disease progression may contribute to the observed structural differences.

Because of the retrospective design, however, the present study cannot distinguish between these potential mechanisms, and the observed associations should therefore not be interpreted as causal.

Descemet membrane endothelial keratoplasty achieved excellent anatomical and functional outcomes in both sexes, consistent with previous reports demonstrating rapid visual rehabilitation, restoration of corneal transparency, favorable endothelial survival, and excellent long-term clinical outcomes after endothelial keratoplasty.^7^, ^8^, ^9^, ^10^, ^11^, ^12^, ^13^ Although male patients demonstrated lower ECC at 12 months in the univariable analysis, this association was no longer statistically significant after adjustment for baseline ECC, donor characteristics, surgical procedure, and baseline CCT. Importantly, donor ECC was an independent determinant of 12-month postoperative ECC (β = 72.6 per 100 cells/mm^2^; *P* = 0.011), and donor-to-postoperative endothelial cell loss did not differ significantly between women and men. Accordingly, the absolute postoperative ECC difference should not be interpreted as strong evidence of inferior endothelial recovery in male patients; rather, postoperative ECC appears to reflect baseline and donor-related factors in addition to recipient sex.

The moderate positive correlation between CCT and ECC observed in male patients (Spearman ρ = 0.483, *P* = 0.027) was an exploratory finding and is biologically counterintuitive. No mechanistic or biological significance should be inferred from this association. Given the number of correlation analyses performed and the absence of multiplicity adjustment, this isolated statistically significant result may represent a chance finding and requires independent confirmation in larger prospective cohorts.

In contrast, biological sex remained independently associated with greater PCT at 12 months after surgery. This finding indicates that regional corneal thickness may represent a more sensitive structural marker of postoperative remodeling than CCT alone.

Although CCT normalized similarly in both sexes, peripheral thickness remained significantly greater in male patients, suggesting persistent regional differences in stromal remodeling or endothelial pump function that are not fully captured by conventional pachymetry.

Future studies should investigate whether these differences have prognostic implications for long-term graft function or disease progression.

Corneal endothelial wound healing after DMEK depends predominantly on enlargement and migration of residual endothelial cells rather than cellular proliferation.^22^, ^23^, ^24^, ^25^, ^26^, ^27^, ^28^ Experimental studies have demonstrated considerable migratory capacity of corneal endothelial cells after injury and endothelial keratoplasty, including migration beyond the immediate graft–host interface.^22^, ^23^, ^24^, ^25^, ^26^, ^27^, ^28^ These biological mechanisms likely contribute to postoperative corneal remodeling and may partly explain why structural recovery extends beyond changes in CCT. Nevertheless, because the present study did not directly evaluate endothelial migration or cellular function, these mechanisms remain speculative.

Recipient-related factors are unlikely to represent the only determinants of postoperative outcome. Previous studies have suggested that donor characteristics may also influence endothelial survival after DMEK.^29^

Although donor age and donor ECC were comparable between female and male patients in the present cohort, additional donor-related variables, including donor sex, preservation time, endothelial resilience, and donor–recipient sex matching, were unavailable for analysis. Furthermore, sex-specific immunological mechanisms have been implicated in transplantation biology and may influence graft survival under certain conditions.^30^ Future multicenter studies incorporating these variables may further clarify their contribution to postoperative corneal remodeling.

The higher prevalence of FECD among women observed in the present cohort is consistent with previous epidemiological studies.^31^ Several mechanisms have been proposed to explain this sex disparity, including hormonal influences, oxidative stress, mitochondrial dysfunction, and metabolic susceptibility.^31^ More recently, ultraviolet A–induced DNA damage has emerged as another potential contributor to endothelial degeneration in FECD.^32^ While these mechanisms may explain the greater prevalence of FECD in women, they do not readily account for the more advanced structural disease observed in male patients in the present study, highlighting the multifactorial nature of FECD pathogenesis.

Rebubbling procedures were required only in female patients; however, this difference did not reach statistical significance and should be interpreted cautiously given the small number of events. Consequently, the present data do not support conclusions regarding sex-specific differences in postoperative graft attachment or complication rates.

Several limitations should be acknowledged. First, the retrospective single-center design limits causal inference and may have introduced selection bias. Of 181 screened eyes, 37 (20.4%) were excluded because of incomplete 12-month follow-up and/or missing clinical data; this relatively large exclusion may have introduced selection and attrition bias if excluded patients differed systematically from the analyzed cohort. Although the screening process and reasons for exclusion are transparently reported in Table 1, residual selection bias cannot be excluded. Second, the relatively small number of male patients reduced statistical power and limited subgroup analyses. Third, standardized clinical grading of FECD severity was not consistently available. Therefore, baseline disease severity was approximated using ECC and corneal thickness, which were also included as covariates in the multivariable regression models. Fourth, the secondary univariable comparisons and correlation analyses were exploratory, and no formal correction for multiple testing was applied. Consequently, isolated statistically significant findings, particularly among the correlation analyses, may reflect chance and should be interpreted cautiously. Finally, endothelial cell density measurements in FECD remain technically challenging because guttae may interfere with automated specular microscopy analysis.^33^ Nevertheless, all examinations were performed using standardized imaging protocols, and repeated measurements were obtained to improve measurement reliability.

Despite these limitations, the present study has several strengths. It represents one of the larger sex-stratified cohorts evaluating structural outcomes after DMEK in FECD and includes comprehensive longitudinal assessment of ECC together with CCT and PCT. Furthermore, the incorporation of multivariable regression analyses allowed adjustment for major confounding variables and provided a more robust assessment of the independent association between biological sex and postoperative structural outcomes.

In conclusion, the present findings suggest that biological sex may be associated with differences in the structural manifestations of FECD before surgery and with postoperative corneal remodeling after DMEK.

While postoperative visual outcomes and CCT were comparable between women and men, male patients exhibited persistently greater PCT throughout follow-up. In contrast, the observed differences in postoperative ECC were largely explained by baseline disease severity and donor-related factors.

These findings support the inclusion of biological sex as an important covariate in future clinical studies and highlight the potential value of structural imaging biomarkers for a more comprehensive assessment of endothelial keratoplasty outcomes.

## Conclusions

This study demonstrates that male patients with FECD presented with a more advanced structural disease phenotype than female patients, characterized by lower ECC and greater CCT and PCT despite comparable preoperative visual acuity. After DMEK, both sexes achieved comparable long-term visual rehabilitation and normalization of CCT. Although male patients exhibited lower postoperative ECC in the univariable analysis, this association was no longer independent after adjustment for baseline disease severity and donor-related factors. In contrast, male sex remained independently associated with greater postoperative PCT. These findings support consideration of biological sex as an important covariate in future clinical studies and highlight the potential value of PCT as a complementary structural biomarker after DMEK. Future prospective multicenter studies incorporating standardized disease severity grading and comprehensive longitudinal endothelial cell analyses are warranted to further elucidate the biological mechanisms underlying these sex-specific differences and their implications for postoperative graft remodeling and long-term clinical outcomes.
